# Transfer Learning: Making Retrosynthetic Predictions Based on a Small Chemical Reaction Dataset Scale to a New Level

**DOI:** 10.3390/molecules25102357

**Published:** 2020-05-19

**Authors:** Renren Bai, Chengyun Zhang, Ling Wang, Chuansheng Yao, Jiamin Ge, Hongliang Duan

**Affiliations:** Artificial Intelligent Aided Drug Discovery Lab, College of Pharmaceutical Science, Zhejiang University of Technology, Hangzhou 310014, China; renrenbai@zjut.edu.cn (R.B.); zcy953268293@163.com (C.Z.); wangling6178@126.com (L.W.); yaochuansheng95@126.com (C.Y.); gejiamin1212@163.com (J.G.)

**Keywords:** retrosynthesis, SMILES structure, reactants, products, transfer leaning, artificial intelligence, transformer, seq2seq

## Abstract

Effective computational prediction of complex or novel molecule syntheses can greatly help organic and medicinal chemistry. Retrosynthetic analysis is a method employed by chemists to predict synthetic routes to target compounds. The target compounds are incrementally converted into simpler compounds until the starting compounds are commercially available. However, predictions based on small chemical datasets often result in low accuracy due to an insufficient number of samples. To address this limitation, we introduced transfer learning to retrosynthetic analysis. Transfer learning is a machine learning approach that trains a model on one task and then applies the model to a related but different task; this approach can be used to solve the limitation of few data. The unclassified USPTO-380K large dataset was first applied to models for pretraining so that they gain a basic theoretical knowledge of chemistry, such as the chirality of compounds, reaction types and the SMILES form of chemical structure of compounds. The USPTO-380K and the USPTO-50K (which was also used by Liu et al.) were originally derived from Lowe’s patent mining work. Liu et al. further processed these data and divided the reaction examples into 10 categories, but we did not. Subsequently, the acquired skills were transferred to be used on the classified USPTO-50K small dataset for continuous training and retrosynthetic reaction tests, and the pretrained accuracy data were simultaneously compared with the accuracy of results from models without pretraining. The transfer learning concept was combined with the sequence-to-sequence (seq2seq) or Transformer model for prediction and verification. The seq2seq and Transformer models, both of which are based on an encoder-decoder architecture, were originally constructed for language translation missions. The two algorithms translate SMILES form of structures of reactants to SMILES form of products, also taking into account other relevant chemical information (chirality, reaction types and conditions). The results demonstrated that the accuracy of the retrosynthetic analysis by the seq2seq and Transformer models after pretraining was significantly improved. The top-1 accuracy (which is the accuracy rate of the first prediction matching the actual result) of the Transformer-transfer-learning model increased from 52.4% to 60.7% with greatly improved prediction power. The model’s top-20 prediction accuracy (which is the accuracy rate of the top 20 categories containing actual results) was 88.9%, which represents fairly good prediction in retrosynthetic analysis. In summary, this study proves that transferring learning between models working with different chemical datasets is feasible. The introduction of transfer learning to a model significantly improved prediction accuracy and, especially, assisted in small dataset based reaction prediction and retrosynthetic analysis.

## 1. Introduction

**Figure 1:**
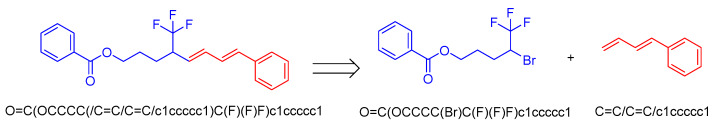
An example of a retrosynthetic prediction. The target module is shown to the left of the arrow and predicted reactants are displayed to the right. The SMILES code for each compound is also indicated.

Organic synthesis is a crucial discipline that predicts accesses to molecules. Two closely related problems contribute to the synthesis of new molecules: forward reaction prediction and retrosynthetic reaction prediction. Forward reaction prediction is inference of the potential products of a given set of reactants, reagents and reaction conditions. The retrosynthetic reaction prediction is the inverse solution to the problem of synthesis. The retrosynthetic reaction prediction starts from the target compounds and transforms them into simpler compounds that are commercially available (Figure 1). Synthetic organic chemistry underpins many areas of chemistry, but the execution of complex chemical syntheses requires expert knowledge usually acquired over many years of study and hands-on laboratory practice [1] Driven by improved computing power, data availability and algorithms, the development of artificial intelligence (AI) technologies with the potential to streamline and automate chemical synthesis is becoming a reality. A few decades ago, computational algorithms could help chemists predict the synthetic routes to some simple target molecules [2]. Although the reaction routes to these compounds can easily be artificially designed without the help of a computer, the use of algorithm models in retrosynthetic prediction has proved to be feasible. However, as computing models become increasingly sophisticated and datasets continue to be enriched, predicting synthetic methods for novel or complex molecules by using AI is no longer an impossible task.

Actually, both simple and complex chemical structures can be treated as a special kind of language that can be recognized by computers. More importantly, the structure and the language should be freely and correctly converted into each other. The simplified molecular-input line-entry system (SMILES), as a computational language, is just a recognizable text sequence inline notation format [3]. Through the transformation of equivalent chemical structures into SMILES codes, several practical sequence models can be successfully applied to reaction prediction. The main models include the sequence-to-sequence (seq2seq) model [4], an encoder-decoder recurrent neural network, and the Transformer model [5], which is an attention-based neural machine translation (NMT) model. The Transformer and seq2seq models were originally designed for natural language translation and some researchers have applied them to the prediction of forward reaction and the prediction of retrosynthetic reaction, respectively [4,5]. Chemical structures can be treated as a special kind of language—specifically, SMILES. Therefore, researchers have innovatively applied the Transformer and seq2seq models to chemical reaction predictions and have regarded prediction tasks as language translation.

Using end-to-end training on a combination of artificially generated reactions, Nam and Kim first introduced the concept of treating chemical reactions as a translation problem [6]. The authors explored forward reaction prediction based on two training sets: one from the patent database and one from the reaction templates in an organic chemistry textbook by Wade [4]. Schwaller et al. applied the seq2seq model to predict the outcomes of complex organic chemistry reactions [7]. Based on a much larger and updated dataset (Lowe’s dataset [6]) and using Luong’s attention mechanism, which is a computer module that can make it easier for neural networks to focus on chemical information such as a reaction center, the prediction accuracy of neural networks has significantly improved. Liu et al. made the first steps toward using the seq2seq model in retrosynthetic analysis [8]. The authors used a dataset containing 50,000 reactions classified into ten different reaction classes. Consequently, the seq2seq model can not only predict the reaction products in the forward reactions but also perform retrosynthetic prediction analysis.

The Transformer, another frequently used model, was proposed by Vaswani [5]. The Transformer is based solely on attention mechanisms, thus dispensing entirely with recurrence and convolutions. Schwaller and Lee’s group successfully applied a Molecular Transformer model to uncertainty-calibrated chemical reaction prediction [9]. Lee also used the Transformer model to unify reaction prediction and retrosynthesis across pharma chemical space [10]. Experiments by the authors on two machine translation tasks showed that the Transformer was superior to the seq2seq model [5].

Machine learning and data mining techniques have been used in numerous real-world applications where the training data and testing data are taken from the same domain or dataset [11]. However, in some real-world scenarios, this approach is difficult to execute because of a lack of useful data or of difficulty in collecting matching data for training [12]. In such cases, transfer learning would be a desirable methodology for a target domain trained on a related source domain.

Transfer learning is a machine learning method in which a model developed for a task is reused as the starting point for a model on a second task, thereby transferring knowledge from a source domain to a target domain; an example of transfer learning is solving one problem and applying it to a different but related problem [13]. Research on transfer learning has attracted increasing attention since 1995 under different names, such as learning to learn, life-long learning, knowledge transfer, inductive transfer, multitask learning, knowledge consolidation and context-sensitive learning [14]. Transfer learning has been successfully applied to many machine learning problems, including text sentiment classification, image classification and multilanguage text classification [15,16,17]. In theory, as a kind of AI technology, transfer learning can also be applied to organic and medicinal chemistry, especially reaction prediction and retrosynthetic analysis based on datasets containing very limited data volume.

Some chemical reaction predictions and retrosynthetic analysis target specific reaction types, such as oxidation reactions involving special oxidants, coupling reactions catalyzed by particular metals and specific ligands catalyzing the activation of hydrocarbons and asymmetric synthesis. Existing datasets often include very few of the above reactions. When the deep learning method is applied to retrosynthetic analysis or predictions of the products of these reactions, it is difficult to obtain accurate prediction results because the dataset is too limited to adequately train the model.

**Figure 2:**
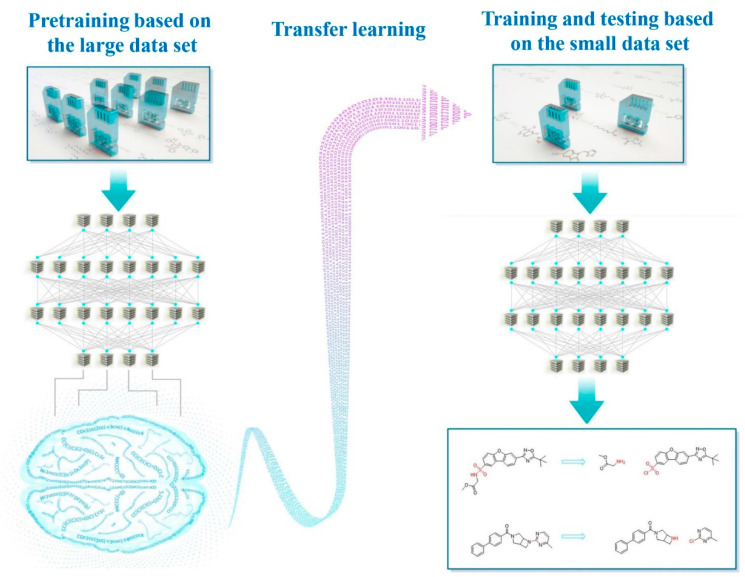
Designing concept and process of transfer-learning-aided retrosynthetic analysis.

Therefore, in this work (Figure 2), to increase the accuracy of retrosynthetic analysis, we introduced the transfer learning strategy to the seq2seq and Transformer models. First, pretraining was performed on a large chemical reaction dataset. After being adequately trained, the models obtained special chemical reaction knowledge. Second, the learned knowledge was successfully transferred to be used on a smaller dataset. Finally, with the chemical skills from the pretraining, the models could output results with increased accuracy after a short and limited training on a small dataset. To compare with the previous results, we deliberately selected the same small dataset as the reference data.

## 2. Methods

### 2.1. Dataset Preparation

The USPTO-380K dataset [6] was first used for the pretraining of the seq2seq and Transformer models. The USPTO-50K dataset [8] was then applied to the corresponding seq2seq-transfer-learning and Transformer-transfer-learning models (data sets are available in the Supplementary Materials).

#### 2.1.1. Pretraining Dataset Preparation: USPTO-380K

The large dataset applied in the pretraining of the seq2seq and Transformer models was named USPTO-380K and included approximately 380,000 examples. The source of the reaction examples was derived from Lowe’s patent mining work, which extracted these openly available reaction examples from the United States Patent and Trademark Office (USPTO) patents granted between 1976 and 2016. The reaction examples were preprocessed to eliminate all reagents, and the reactants and products were maintained without dividing them into ten classes, as Liu did [8]. Moreover, the duplicate, incomplete or erroneous reactions were filtered as noisy data. Additionally, for the testing dataset, the reactions in Liu’s dataset (USPTO-50K) were removed from our USPTO-380K dataset.

#### 2.1.2. Small Dataset Preparation: USPTO-50K

The small dataset we used in this paper is USPTO-50K and is applied to seq2seq-transfer-learning and Transformer-transfer-learning models. This dataset contains 50,000 reaction examples and was also used by Liu et al. [8]. The source of the dataset is USPTO patents prepared by Lowe [6]. The dataset was subsequently split into training, validation and test datasets (8:1:1). As shown in Table 1, these 50,000 reactions were classified mainly into 10 reaction types. The contextual information, including reagents, temperature and reaction time, was further eliminated so that the reaction examples included only starting materials and their corresponding products.

### 2.2. Models

#### 2.2.1. Seq2seq Model

The seq2seq model is based on an encoder-decoder architecture that consists of two distinct recurrent neural networks (RNNs) working cooperatively [19]. The encoder addresses the input sequence and then exports the corresponding context vector to the decoder. The decoder applies this representation to pass a set of predictions. These two RNNs consist of long short-term memory (LSTM) cells, which efficiently dispose of long-range relations in sequences [20]. This architecture has a bidirectional LSTM (BLSTM) encoder and an LSTM decoder [21]. The BLSTM encoder consists of two LSTM cells in which one handles the sequence in the forward direction and the other handles the sequence in the backward direction. In this sense, the BLSTM processes input sequences in two directions and it possesses context from not only the past but also the future. Moreover, an additive attention mechanism that connects the target inputs with the source outputs is applied to this model [22]. Molecules can be equivalently represented as a sequence of SMILES. From a linguistic point of view, SMILES and a chemical reaction can be regarded as separate languages. Therefore, predicting the corresponding chemical reaction can be regarded as a language translation task. The seq2seq model is a language translation model, so this model can be applied to chemical reaction prediction.

#### 2.2.2. Transformer Model

The Transformer model, which is based on an encoder-decoder architecture, shows state-of-the-art performance in chemical reaction prediction and retrosynthetic analysis [9,23,24]. The architectural feature of the Transformer model completely relies on the attention mechanism. The encoder consists of several identical layers, and each layer contains two different sublayers: the first sublayer is a multi-head self-attention mechanism, and the second is a position feedforward network. Before layer normalization, a residual connection to the two sublayers is commonly introduced. The decoder consists of six identical layers, and each layer has three sublayers. In addition to two sublayers that are the same as those in the encoder, a third layer, a masked multi-head self-attention mechanism, is added to the decoder. Remarkably, a major feature of the Transformer model (multi-head attention) makes the Transformer model superior to the seq2seq model by allowing the Transformer model to concurrently attend to various representations [5]. First, the chemical reactions were converted into SMILES for training the Transformer model. The SMILES of the target compounds were then input into the model, and the Transformer model output the reactant SMILES.

### 2.3. Performance Evaluation

Top-1 means that the predicted results by the model scan stops as soon as the first prediction is found. Top-2 means that the predicted results by the model scan stops as soon as the first two predictions are found. In top-N, the “N” represents all positive integers. Namely, the top-N could be top-1, top-2, top-3 and so on. Top-N means that the predicted results by the model scan stops as soon as the first N predictions are found. The top-N accuracy refers to the percentage of the ground truth reactant set that was found within the top N predictions made by the model. The calculation formula is as follows:

Top-N Accuracy = (right predictions)/(ground truth) × 100%

The accuracy is estimated for the classification problem in our prediction task. True positives (TPs) are results that match the ground truth and have a confidence score above the threshold, and true negatives (TNs) are results that do not match the ground truth and have a confidence score below the threshold. Results that match the ground truth but have a confidence score below the threshold are counted as false negatives (FNs), and results that do not match the ground truth but have a confidence score above the threshold are regarded as false positives (FPs). 

## 3. Results and Discussion

**Figure 3:**
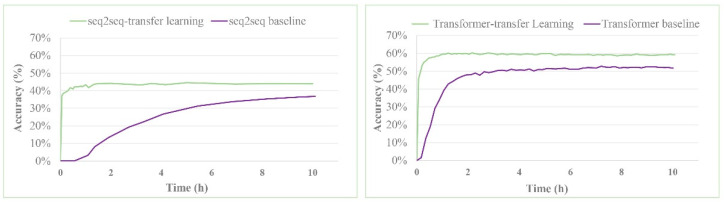
The top-1 accuracies of the seq2seq/seq2seq-transfer-learning and Transformer/Transformer-transfer-learning models as a function of time.

The top-N accuracy (especially the top-1 accuracy), which refers to the percentage of examples in the ground truth reactant set, is usually used as a key measure of the validity of a model. These examples are the actual patent literature-reported reactant set for the corresponding target molecule in the test dataset, which is found within the top-N predictions made by the model. Figure 3 shows the influence of training time on the top-1 results. With the help of the chemical skills acquired in the pretraining, models utilizing transfer learning obtained the “chemical talent” at the very beginning and were no longer “naive” like the single seq2seq or Transformer model. The transfer-learning-based models were very highly top-1 accurate from the start of the training; thus, at this point, their accuracy was already similar to the highest accuracy achieved. The top-1 accuracy of the seq2seq and Transformer models increased slowly during training; these models achieved the highest levels and reached a plateau in approximately 10 hours. The transfer-learning-based, seq2seq-transfer-learning and Transformer-transfer-learning models were markedly more accurate than the corresponding single seq2seq or Transformer models.

A comparison of the top-N accuracies of the seq2seq-transfer-learning model and Liu’s seq2seq model [8] is shown in Table 2. With the introduction of the transfer learning strategy, the accuracy of the seq2seq-transfer-learning model in retrosynthetic analysis displayed a significant improvement over that of the seq2seq baseline. Moreover, this improvement increased steadily. The top-1, top-3, top-5, top-10 and top-20 accuracies increased between 6.2% and 7.2%. For instance, the accuracy of top-1 increased from 37.4% to 44.6%, an improvement of 7.2%; the accuracy of top-5 increased from 57.0% to 64.1%, an improvement of 7.1%; and the accuracy of top-20 increased from 65.9% to 72.1%, an improvement of 6.2%. The obtained data also clearly demonstrated that when the chemical knowledge learned from the pretraining was transferred to the test model, the retrosynthetic analysis results were more accurate than the results obtained without transfer learning.

Table 3 shows the performance of the Transformer and Transformer-transfer-learning models. These results indicate that for retrosynthetic analysis, the accuracy of the Transformer model was remarkably superior to that of the seq2seq model. According to our calculation results, the accuracy of the Transformer-transfer-learning model ranged from being 8.3% to 14.6% higher than the accuracy of the Transformer baseline. The top-1 accuracy of the Transformer-transfer-learning model was 60.7%, which represented state-of-the-art performance based on an open-source patent database containing 50,000 reaction examples [6]. The top-20 accuracy of the model increased to 88.9%, which is an excellent result in retrosynthetic analysis. Table 4 shows TPs, TNs, FPs and FNs in top-1 predictions after transfer learning in transformer model. The F_1_-score [25], a statistical measure, is used to rate performance as a weighted harmonic mean of precision and recall. Here are the relevant calculation formulae.

Precision = TP/(TP + FP)

Recall = TP/(TP + FN)

F_1_ = (2∙Precision∙Recall)/(Precision + Recall)

In our experiment, the F_1_-score of top-1 predictions by the Transformer-transfer-learning model can reach 67.3% which reveals good performance of our model. Apart from the real accuracy we used, delta_ accuracy is also critical for estimating model improvement. The delta_ accuracy [25,26] is calculated by the formula Delta_accuracy = Accuracy − [(TP + FN)(TP + FP) + (TN + FN)(TN + FP)] / (N ^ 2)). In the case of contingency table values given in Table 4, delta_ accuracy is 9.0%, which is good and significant result. 

**Figure 4:**
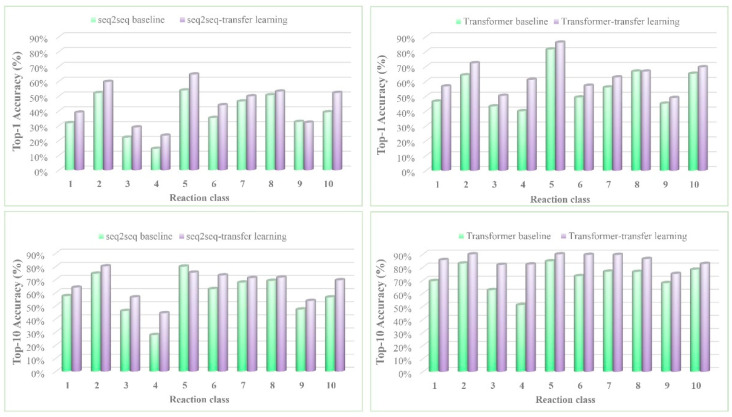
The top-1 and top-10 accuracies of the seq2seq/seq2seq-transfer-learning and Transformer/Transformer-transfer-learning models by reaction class. 1. Heteroatom alkylation and arylation. 2. Acylation and related processes. 3. C−C bond formation. 4. Heterocycle formation. 5. Protections. 6. Deprotections. 7. Reductions. 8. Oxidations. 9. Functional group interconversion (FGI). 10. Functional group addition (FGA).

Figure 4 illustrates the detailed top-1 and top-10 accuracies for the 10 reaction classes. In terms of the top-1 results, the seq2seq-transfer-learning models performed significantly better than the seq2seq model for reaction classes 1 (heteroatom alkylation and arylation, with 10.2% improvement) and 4 (heterocycle formation, with a significant 21.1% increase). Moreover, the Transformer-transfer-learning model demonstrated favorable results (a 21.1% increase in accuracy) for reaction class 4.

When the performance is based on the top-10 results, the Transformer-transfer-learning model was very highly accurate for reaction classes 3 (C−C bond formation, with a significant increase of 19.3%) and 4 (with a remarkable 31.1% increase); these results are significantly better than those of the other three models. However, the seq2seq-transfer-learning model is slightly more accurate than seq2seq.

The above results all proved that introducing the transfer learning strategy, especially to the Transformer model, significantly improved the accuracy of retrosynthetic analysis. Because the Transformer-transfer-learning model effectively understood and used the chemical knowledge gained from the pretraining, introducing transfer learning was very helpful in increasing the accuracy of predictions, even for some small datasets. For example, reaction class 4 contains only 900 reactions, but the top-1 and top-10 accuracies of the Transformer-transfer-learning model were remarkably more accurate than those of the Transformer model alone by 21.1% and 31.1%, respectively.

As illustrated in Table 5, the accuracy of the top-1 prediction by the Transformer model for heterocycle formation reactions (class 4) was significantly lower (by only approximately 40%) than the accuracy of the top-1 prediction for other reaction types. This difference in accuracy is mainly because the ring-forming reaction itself is very complicated, and the prediction is fairly difficult. Moreover, in the heterocycle formation reactions, the SMILES codes underwent a complex transformation between the linear and cyclic structures, thereby significantly increasing the probability of errors. All of these factors made this prediction accuracy the lowest among those for the 10 types of reactions. However, with the help of pretraining, the Transformer-transfer-learning model can identify the chemical structures with increased accuracy; e.g., the accuracy of the top-1 prediction remarkably increased by 21.1% to 61.1%.

The Transformer model displayed limited understanding and identification of nonaromatic rings containing heteroatoms, especially spiro and bridged ring structures. In the conversion of SMILES codes and chemical structures in the retrosynthetic analysis, corresponding errors in ring structures occurred frequently (Table 6). For example, the structure of compound 4 contains a 7-membered ring lactam skeleton. However, the structure of the raw material predicted by the Transformer model is a 9-membered ring lactam. In terms of compound 6, the (*1S*)-8-azabicyclo [3.2.1] octane in its structure was incorrectly recognized as (*6R*)-2-azabicyclo [4.2.0] octane. For all seven error examples produced by the Transformer model in Table 6, the Transformer-transfer-learning model could successfully predict the correct structures of the starting materials, thereby demonstrating the high superiority of this model.

Another common deficiency of the Transformer model in retrosynthetic prediction was the misidentification of chiral structures. As shown in Table 7, when the target compounds contained one or more chiral carbons, although the Transformer model predicted the chemical structure of the raw material, the model often produced a stereo configuration error, in which the stereo configuration of the corresponding *R* or *S* enantiomers was misidentified. The Transformer-transfer-learning model understood chirality better than the Transformer model alone and successfully identified the exact structure of the enantiomer. In the prediction of target compound 3, although the chlorine-atom-bonded carbon atom is in the *S* configuration, the raw material predicted by the Transformer model was racemic. However, the Transformer-transfer-learning model accurately identified this carbon atom as having the *S* configuration.

Consistent with our previous research findings [23], when the target compound contained a *tert*-butyl moiety, SMILES code and prediction errors usually occurred in the Transformer model in the prediction process. As shown in Table 8, the prediction of target compounds 1 and 2 did not obtain any reactant due to errors in the SMILES codes; these errors occurred frequently in the whole prediction for all reaction classes. For the target compounds 3-6, the predicted reactant structures were far from the desired results, and the target compounds were completely impossible to synthesize with the starting materials. However, after pretraining based on the large dataset, the Transformer-transfer-learning model had a highly improved ability to recognize and translate the structures of the compounds, thereby greatly reducing the negative effect of the *tert*-butyl structure on the prediction results and significantly improving the accuracy of the retrosynthetic analysis.

## 4. Conclusions

In summary, the pretraining phase of the Transformer-transfer-learning model increases the ability of the model to accurately understand the complex structural characteristics of compounds and accumulate chemical knowledge related to organic reactions. More importantly, this model can successfully transfer learned chemistry knowledge and skills to a new retrosynthetic prediction model, thereby significantly improving the accuracy of predictions which translate products SMILES to reactants SMILES, and the quality of results.

To facilitate comparisons and verify the value of transfer learning, the USPTO-50K dataset was used for prediction and verification. However, in fact, the transfer learning strategy can be flexibly applied to additional available or self-built small datasets for retrosynthetic analysis. Hypothetically, in addition to retrosynthetic prediction, transfer learning can also be used for reaction prediction. Our future work will focus on the application of transfer learning to certain name reactions. Predicting the outcomes of complex name reactions involving regioselectivity and site-selectivity is an extremely difficult mission even for experienced chemists. This means that this area of research could benefit from AI prediction tools.

## Figures and Tables

**Table 1:** Distribution and description of the major reaction classes within the processed reaction dataset [18].

Class	Description	No. of Examples	Percentage of Dataset (%)
**1**	heteroatom alkylation and arylation	15122	30.3
**2**	acylation and related processes	11913	23.8
**3**	C−C bond formation	5639	11.3
**4**	heterocycle formation	900	1.8
**5**	protection	650	1.3
**6**	deprotection	8353	16.5
**7**	reduction	4585	9.2
**8**	oxidation	814	1.6
**9**	functional group interconversion (FGI)	1834	3.7
**10**	functional group addition (FGA)	227	0.5

**Table 2:** Comparison of the top-N accuracies ^a^ of the seq2seq and seq2seq-transfer learning models.

Model	Top-N Accuracy (%)					
	Top-1	Top-2	Top-3	Top-5	Top-10	Top-20
**seq2seq baseline ^b^**	37.4%	--	52.4%	57.0%	61.7%	65.9%
**seq2seq-transfer learning**	44.6%	54.8%	59.4%	64.1%	68.8%	72.1%

^a^ Data are related to the test data set containing 5004 reactions. ^b^ The testing results are from the seq2seq2 model of Liu et al. [8].

**Table 3:** Comparison of the top-N accuracies ^a^ of the Transformer and Transformer-transfer-learning models.

Model	Top-N Accuracy (%)					
	Top-1	Top-2	Top-3	Top-5	Top-10	Top-20
**Transformer baseline**	52.4%	63.3%	67.1%	70.8%	73.2%	74.3%
**Transformer-transfer learning**	60.7%	74.0%	79.4%	83.5%	87.6%	88.9%

^a^ Data are related to the test data set containing 5004 reactions.

**Table 4:** True positives (TPs), true negatives (TNs), false positives (FPs) and false negatives (FNs) in top-1 predictions ^a^ by the Transformer-transfer-learning model.

	Positive (exp.)	Negative (exp.)
**Positive (pred.)**	2021	776
**Negative (pred.)**	1191	1016
**Total**	5004	

^a^ Data are related to the test data set (N = 5004 reactions).

**Table 5:** Comparisons and representative examples (selected from the test set) of the Transformer and Transformer-transfer-learning models in the retrosynthetic prediction of heterocycle formation reactions.

	Target Compound	Retrosynthetic Analysis	
		Transformer Model (Incorrect Prediction)	Transformer-Transfer-Learning Model (Correct Prediction)
**1**	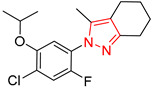	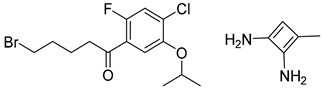	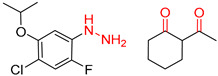
2	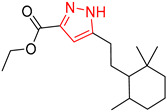	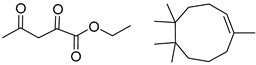	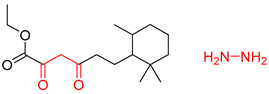
3	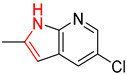	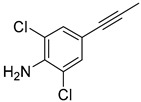	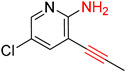
4	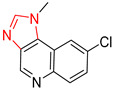	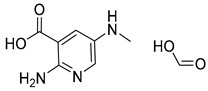	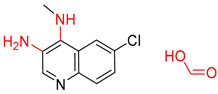
5	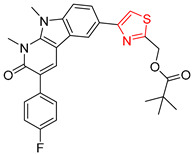	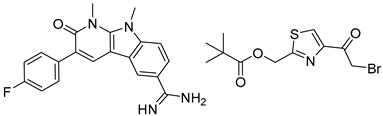	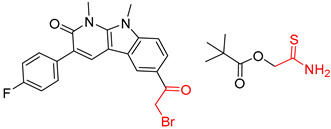

**Table 6:** Comparisons and representative examples (selected from the test set) of the Transformer and Transformer-transfer-learning models in retrosynthetic prediction with nonaromatic heterocycle structures.

	Target Compound	Retrosynthetic Analysis	
		Transformer Model (Incorrect Prediction)	Transformer-Transfer-Learning Model (Correct Prediction)
**1**	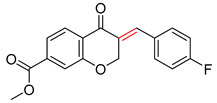	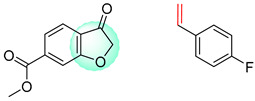	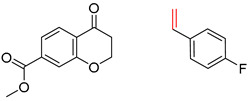
2	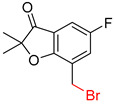	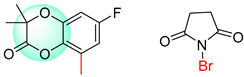	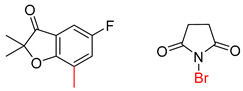
3	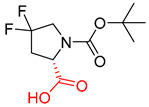	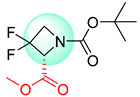	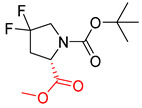
4	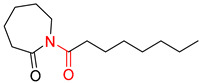	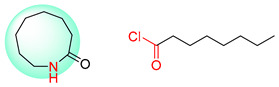	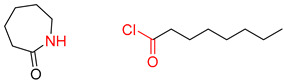
5	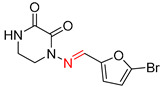	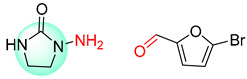	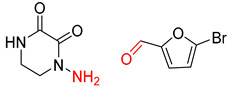
6	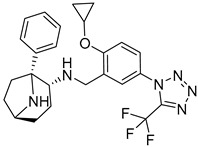	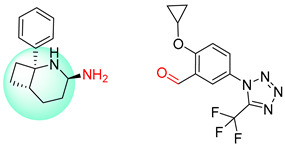	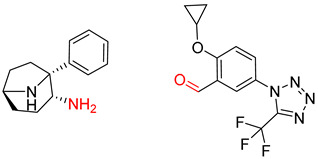
7	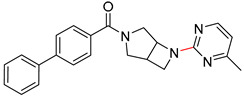	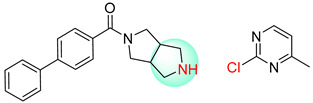	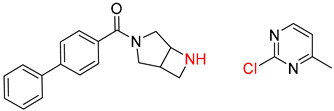

**Table 7:** Comparisons and representative examples (selected from the test set) of the Transformer and Transformer-transfer-learning models in retrosynthetic prediction with chiral carbon atoms.

	Target Compound	Retrosynthetic Analysis	
		Transformer Model (Incorrect Prediction)	Transformer-Transfer-Learning Model (Correct Prediction)
**1**	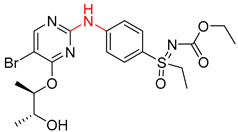	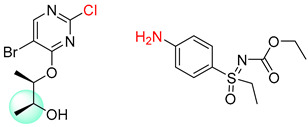	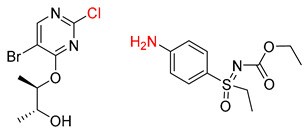
2	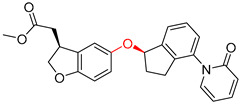	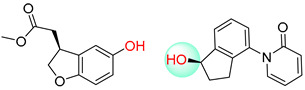	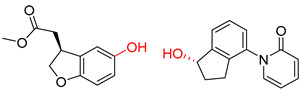
3	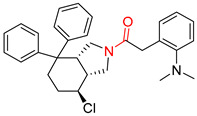	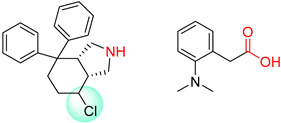	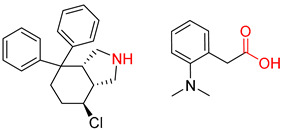
4	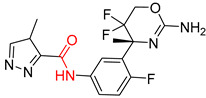	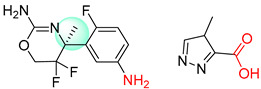	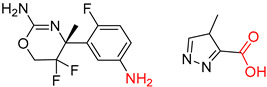
5	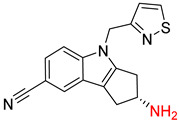	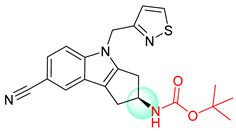	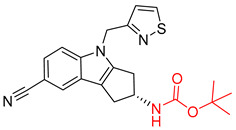
6	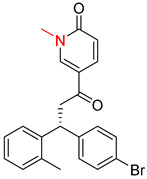	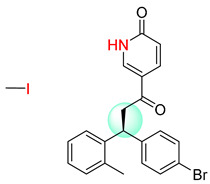	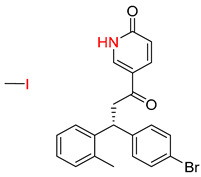
7	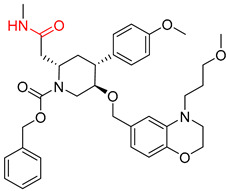	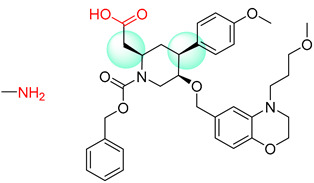	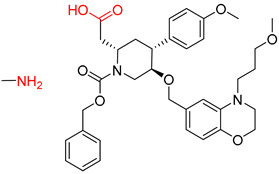

**Table 8:** Comparisons and representative examples (selected from the test set) of the Transformer and Transformer-transfer-learning models in retrosynthetic prediction with tert-butyl moieties.

	Target Compound	Retrosynthetic Analysis	
		Transformer Model (Incorrect prediction)	Transformer-Transfer-Learning Model (Correct Prediction)
**1**	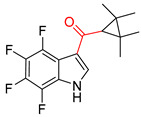	SMILES code error	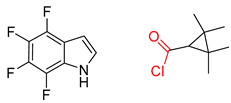
2	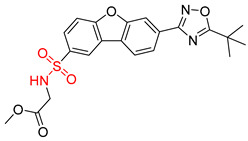	SMILES code error	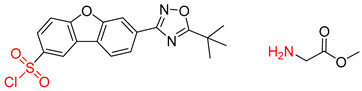
3	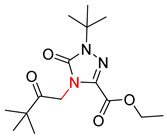	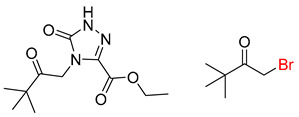	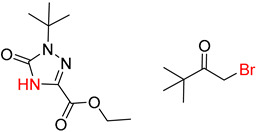
4	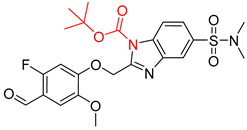	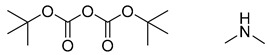	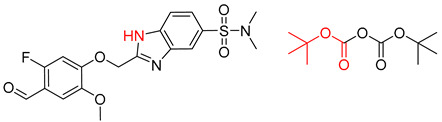
5	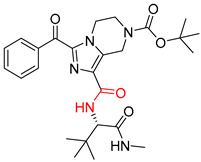	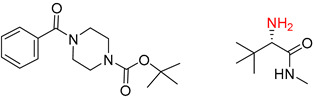	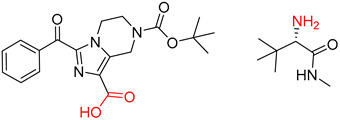
6	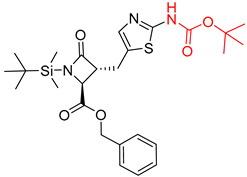	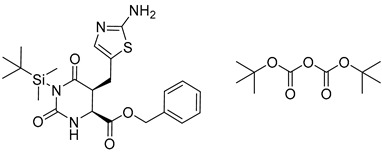	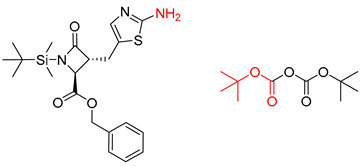

## Supplementary Materials

Training, validation and test sets (SMILES structures of reactants and products including reaction class designation) used in modelling are given.
